# Analysis of Angiographic Patterns in Acute Coronary Syndrome Patients With Diabetes Mellitus: Correlation With HbA1c Levels

**DOI:** 10.7759/cureus.72028

**Published:** 2024-10-21

**Authors:** Yug Garg, Arun Prabhu Marthandam Manickandan, Hana Khan Ghori, Sushmitha Rameshbabu, Imaduddin Mohammed, Saeed Mohamed, Hussain Mustafa Chiniwala, Saad Mohamed, Bhavya Doshi, Shoraf Pascal

**Affiliations:** 1 Endocrinology Diabetes Medicine, Samatvam Diabetes Endocrinology and Medical Center, Bangalore, IND; 2 Internal Medicine, Central America Health Sciences University Belize Medical College, Ladyville, BLZ; 3 Internal Medicine, JSS Medical College, Mysore, IND; 4 Internal Medicine, Madras Medical College, Chennai, IND; 5 Internal Medicine, Shadan Institute of Medical Sciences, Chennai, IND; 6 Internal Medicine, Mysore Medical College, Mysore, IND; 7 Medicine, Sahyadri Hospital Hadapsar, Chennai, IND; 8 Psychiatry, Kanachur Institute of Medical Sciences, Mangalore, IND; 9 Community and Family Medicine, Swaminarayan Institute of Medical Science and PSM Hospital, Kalol, IND; 10 Community Medicine, Madha Medical College and Research Institute, Chennai, IND

**Keywords:** acute coronary syndrome, angiographic patterns, coronary artery disease, diabetes mellitus, glycated hemoglobin

## Abstract

Background

The relation of diabetes mellitus with cardiovascular diseases is well-known, even acute coronary syndrome (ACS). High levels of glycated haemoglobin (HbA1c) are used is a sign of long-term glycemic control and may be associated with severity in coronary artery disease (CAD). In this particular setting, the aim of this investigation was to examine the relationship between the HbA1c values and the angiographic patterns of patients who were admitted with an ACS diagnosis.

Methodology

A cross-sectional study included 120 patients diagnosed with ACS. The criteria for eligibility included patients with ST-elevation myocardial infarction, unstable angina, and non-ST elevation myocardial infarction with history of documented diabetes mellitus. Every patient had a suitable clinical examination, had their HbA1c checked, and had coronary angiography to determine the severity of their CAD. Descriptive statistics and ANOVA were performed for statistical analyses to determine the connection between angiographic patterns and HbA1c.

Results

Patients with elevated HbA1c levels demonstrated a strong association with severe coronary artery disease. Notably, those with HbA1c exceeding 10.5% exhibited significant triple vessel disease and Type C lesions, indicative of advanced coronary artery disease. Statistical analyses revealed a marked difference in angiographic patterns across various HbA1c categories (p < 0.05).

Conclusion

The findings of this study suggest that maintaining optimal HbA1c levels is essential for mitigating the severity of coronary artery disease in patients with ACS. Moreover, effective glycemic control may be protective against advanced coronary atherosclerosis and subsequent cardiovascular complications in both diabetic and non-diabetic individuals.

## Introduction

Diabetes, a metabolic and endocrine disorder, is emerging as a significant global public health crisis. The prevalence and incidence rates have been steadily increasing in recent decades, making it one of the four major non-communicable diseases prioritized for global action [1,2]. Despite achieving acceptable fasting blood sugar levels, the long-term outlook for diabetes patients can be compromised by inadequate overall management [3]. Glycated hemoglobin (HbA1c) is a valuable indicator of hyperglycemia and glucose intolerance. Unlike fasting blood sugar tests, HbA1c does not require fasting and provides a more comprehensive picture of blood sugar control [4]. Also, compared to fasting glucose levels, it is more dependable. A higher risk of microvascular and macrovascular problems is associated with elevated HbA1c levels [5]. Several studies have shown that the risk of microvascular problems in both type 1 and type 2 diabetes is much lower when optimum glycemic control is maintained, which is defined as HbA1c values ≤7% [6]. Furthermore, regardless of fasting glucose levels, current research indicates that elevated HbA1c levels are predictive of cardiovascular disease and mortality even in those without diabetes [7,8]. Additionally, HbA1c may offer predictive information about potential cardiovascular disease. Research has consistently shown a positive correlation between elevated HbA1c levels and mortality as well as subclinical cardiovascular disease in non-diabetic individuals [9]. Prior to 1980, diabetes and cardiovascular disease were treated as distinct conditions; however, Seegen et al. demonstrated a stronger association between the two [10].

Precise diagnosis is essential for determining the appropriate course of treatment and forecasting results for those suffering from acute coronary syndrome (ACS). Patients often present with chronic chest pain or breathing issues, particularly in the context of long-standing diabetes, which can render their symptoms silent [10]. Invasive procedures like coronary angiography are necessary for confirming coronary artery heart disease (CAHD), assessing the severity of stenosis, and determining the extent of arterial involvement. Non-invasive techniques like resting ECG, stress tests (TMT), echocardiography, and stress thallium imaging are also helpful in diagnosing myocardial ischemia. By providing alternatives for both the diagnosis and treatment of coronary artery disease, "interventional cardiology" has completely changed the discipline [11,12]. The most reliable method for identifying and evaluating coronary artery disease is still coronary angiography. Very few studies have considered the relationship between HbA1c levels and diabetic angiographic findings, despite the fact that numerous investigations have reported angiographic differences in the pattern and the severity of disease between individuals with and without diabetes. Moreover, additional parameters could enhance the comparison of results and discuss mortality benefits associated with coronary angiography, particularly regarding early detection of atherosclerosis in coronary arteries depending on HbA1c levels [10]. In order to assess any potential link between blood levels of HbA1c and angiographic patterns, this study was planned to quantify HbA1c in patients who were admitted with acute coronary syndrome. Despite extensive research demonstrating significant disparities in angiographic patterns and disease severity between diabetic and non-diabetic individuals, few studies have explored the direct correlation between HbA1c levels and angiographic findings in diabetic patients. To elucidate any potential association between HbA1c and angiographic patterns, this investigation was designed to quantify HbA1c levels in patients presenting with acute coronary syndrome.

## Materials and methods

Study design and setting

This was a cross-sectional study conducted in the Department of General Medicine. Patients diagnosed with ACS within the General Medicine and Cardiology departments in the hospital setting formed the population. Consent was obtained or waived by all participants in this study. Madha Medical College and Research Institute issued approval MMCRI/IEC/2022/027.

Selection Criteria

This study included patients diagnosed according to the universal definition of myocardial infarction 2012 as having ST elevation myocardial infarction (STEMI), patients with unstable angina (UA) and non-ST elevation myocardial infarction (NSTEMI) according to relevant criteria for these conditions and patients with a confirmed diagnosis of diabetes mellitus (DM). The diagnosis of diabetes was recorded through medical history, previous laboratory results that showed the establishment of DM, or when patients were clinically diagnosed by a physician as diabetic prior to presentation. This included patients receiving oral antidiabetic medications, insulin therapy, or dietary interventions as per the 2017 Revised Clinical Practice Guidelines of the American Diabetes Association. DM was diagnosed based on the following criteria: fasting plasma glucose (FPG) ≥126 mg/dl, two-hour plasma glucose ≥200 mg/dl during an oral glucose tolerance test (OGTT), HbA1c >6.5%, or the presence of diabetes symptoms with a random plasma glucose ≥200 mg/dl. Patients with severe iron or vitamin B12 deficiency, those on iron or vitamin B12 supplements, patients with chronic liver illness, and patients with chronic renal disease (glomerular filtration rate (GFR) <90 ml/min/1.73 m2) were among the exclusion criteria.

Assessment of Biochemical Parameters

In addition to assessing HbA1c levels, blood samples were collected to evaluate the following parameters: total cholesterol, low-density lipoprotein (LDL), high-density lipoprotein (HDL), triglycerides, and C-reactive protein. These parameters were measured to further investigate their association with the severity of coronary artery disease (CAD) and their relationship with angiographic findings. The results were categorized based on normal coronary arteries, single-vessel disease (SVD), double-vessel disease (DVD), and triple-vessel disease (TVD), and were analyzed in relation to different HbA1c levels.

Additional Data on Duration of T2DM and CAD

To further assess the impact of chronic disease duration, information regarding the duration of T2DM and the duration of CAD was collected through patient interviews and medical records. The duration of T2DM was defined as the number of years since the initial diagnosis, and the duration of CAD was determined based on the time since the first clinical or angiographic diagnosis of coronary artery disease. This data was analyzed to determine its influence on the severity of CAD.

Sample Size Calculation

The sample size for this study was calculated based on previous research, which indicated that 24% of patients with TVD had elevated HbA1c levels, as reported by Hegde et al. [10]. The power calculation was done to detect, at 80%, a difference in the proportion of HbA1c levels between TVD SVD and DVD groups, which we had estimated to be about 20%. Using these parameters, it was calculated that there should be a minimum of 100 participants. With regard to the almost 15% loss in follow-up with some participants, 20 additional participants have been added. This increases the final sample size to 120 participants. This should suffice for the study to provide enough power and precision with its findings.

Data sources and variables

All participants underwent a comprehensive medical history and physical examination. Following informed consent, blood samples were obtained for the measurement of HbA1c and other routine laboratory investigations. Coronary angiographic patterns were meticulously evaluated to assess the extent of coronary artery disease. Participants were subsequently categorized into four groups based on their HbA1c levels: <6.5%, 6.5-8.5%, 8.5-10.5%, and >10.5%. These groups were then compared with coronary angiographic classifications of normal coronary arteries, SVD, DVD, and TVD. The severity of coronary lesions was determined using the American Heart Association (AHA) classification system, which categorizes lesions into Types A, B, and C.

Statistical analysis

The primary outcomes of the study were HbA1c levels and coronary angiography results, while secondary explanatory variables included age, gender, smoking status, alcohol consumption, duration of type 2 diabetes mellitus (T2DM), and CAD. Biochemical parameters such as total cholesterol, LDL, HDL, triglycerides, and C-reactive protein were also analyzed for their association with the severity of CAD. Descriptive statistics were employed, with quantitative variables expressed as means and standard deviations, and categorical variables as frequencies and proportions. Relationships between HbA1c levels, CAD severity, and explanatory variables were analyzed using analysis of variance (ANOVA) for quantitative comparisons, and Chi-square or Fisher's exact test for categorical comparisons. A p-value of less than 0.05 was considered statistically significant. IBM SPSS version 22 was used for all statistical analyses (IBM Corp., Armonk, NY, USA) [13].

## Results

Table 1 below summarizes the demographic profile of the study participants. The average age of patients was 57.6 years, with males forming 78 (65%) of the group. A significant percentage of participants were smokers (48, 40%) and alcohol users (42, 35%), while 54 (45%) had a history of diabetes. The average duration of T2DM among the known diabetics was 8.5 ± 3.2 years, and the average duration of CAD was 4.7 ± 2.1 years.

**Table 1:** Demographic and clinical characteristics T2DM: Type 2 Diabetes Mellitus, CAD: Coronary Artery Disease

Characteristics	N (%)
Total Patients	120
Mean Age (years)	57.6 ± 9.5
Gender (Male)	78 (65%)
Smokers	48 (40%)
Alcohol Users	42 (35%)
Known Diabetics	54 (45%)
Average Duration of T2DM (years)	8.5 ± 3.2
Average Duration of CAD (years)	4.7 ± 2.1

**Figure 1:**
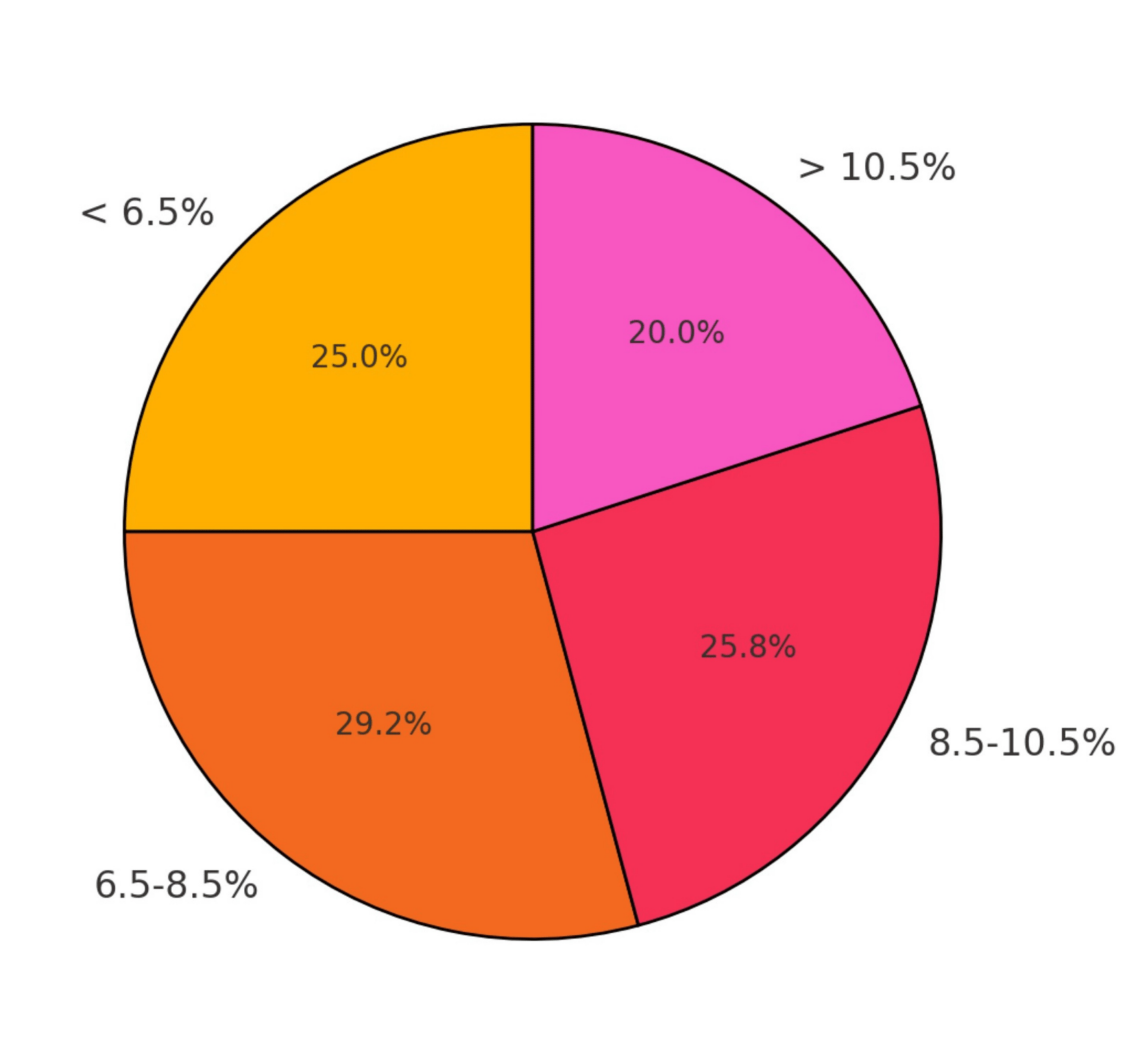
Pie chart showing distribution of HbA1c levels HbA1c: Glycated hemoglobin

The patients were categorized based on HbA1c levels, as presented in Figure 1. Interestingly, a higher proportion of the patients had elevated HbA1c levels (>10.5%), showing a potential association between poor glycaemic control and cardiovascular disease severity.

The coronary angiography (CAG) findings are detailed in Table 2. Patients with higher HbA1c levels, particularly those above 10.5%, were more likely to have TVD. Conversely, those with lower HbA1c levels tended to show normal coronaries or less severe involvement such as SVD.

**Table 2:** Angiographic pattern

Angiographic Pattern	N (%)
Normal Coronaries	24 (20%)
Single Vessel Disease (SVD)	36 (30%)
Double Vessel Disease (DVD)	34 (28.3%)
Triple Vessel Disease (TVD)	26 (21.7%)

Table 3 shows the distribution of angiographic patterns across different HbA1c levels. Among patients with HbA1c >10.5%, 10 (41.7%) presented with TVD, while only one (3.3%) in the HbA1c <6.5% group had TVD. The ANOVA revealed a significant difference in the angiographic severity across different HbA1c groups (p < 0.05).

**Table 3:** Association between HbA1c levels and angiographic pattern *The p-value came out to be less than 0.05 which was considered significant. HbA1c: Glycated hemoglobin, SVD: Single Vessel Disease, DVD: Double Vessel Disease, TVD: Triple Vessel Disease

HbA1c Levels (%)	Normal Coronaries (N = 24)	SVD (N = 36)	DVD (N = 34)	TVD (N = 26)	p-value
< 6.5%	10 (33.3%)	12 (40%)	7 (23.3%)	1 (3.3%)	0.019*
6.5-8.5%	8 (22.9%)	11 (31.4%)	9 (25.7%)	7 (20%)	
8.5-10.5%	4 (12.9%)	9 (29%)	10 (32.2%)	8 (25.8%)	
> 10.5%	2 (8.3%)	4 (16.7%)	8 (33.3%)	10 (41.7%)	

The severity of coronary lesions classified into Types A, B, and C, as per the AHA, is shown in Table 4. Patients with higher HbA1c levels were more likely to have Type C lesions, which are considered more complex and severe. Among patients with HbA1c >10.5%, 11 (45.8%) had Type C lesions. Fisher's exact test revealed a statistically significant association (p < 0.05).

**Table 4:** Severity of lesions based on American Heart Association (AHA) classification *The p-value came out to be less than 0.05 which was considered significant.

HbA1c Levels (%)	Type A Lesions	Type B Lesions	Type C Lesions	p-value
< 6.5%	18 (60%)	10 (33.3%)	2 (6.7%)	0.028*
6.5-8.5%	12 (34.3%)	16 (45.7%)	7 (20%)	
8.5-10.5%	9 (29%)	14 (45.2%)	8 (25.8%)	
> 10.5%	5 (20.8%)	8 (33.3%)	11 (45.8%)	

The study analyzed the association between additional clinical parameters and the severity of CAD, using ANOVA for continuous variables and Fisher's exact test for categorical variables as shown in Table 5. Patients with TVD had significantly higher total cholesterol (230 ± 35 mg/dL), LDL (150 ± 25 mg/dL), triglycerides (240 ± 30 mg/dL), and C-reactive protein (4.0 ± 0.9 mg/L) levels compared to those with normal coronaries, with p-values of <0.001 for each. In contrast, HDL levels were significantly lower in TVD patients (35 ± 5 mg/dL) compared to those with normal coronaries (50 ± 10 mg/dL, p = 0.005). Furthermore, the prevalence of comorbidities such as hypertension and obesity increased with CAD severity, with 57.7% of TVD patients having comorbidities compared to 20.8% of those with normal coronaries (p = 0.004). These findings demonstrate a clear correlation between elevated lipid profiles, inflammatory markers, comorbidities, and the severity of CAD.

**Table 5:** Association of additional parameters with severity of coronary artery disease (CAD) SVD: Single Vessel Disease, DVD: Double Vessel Disease, TVD: Triple Vessel Disease, CRP: C-reactive Protein, LDL: Low-Density Lipoprotein, HDL: High-Density Lipoprotein

Parameter	Normal Coronaries (N = 24)	SVD (N = 36)	DVD (N = 34)	TVD (N = 26)	p-value
Total Cholesterol (mg/dL)	170 ± 30	190 ± 25	210 ± 40	230 ± 35	<0.001*
LDL (mg/dL)	90 ± 20	110 ± 15	130 ± 30	150 ± 25	<0.001*
HDL (mg/dL)	50 ± 10	45 ± 5	40 ± 8	35 ± 5	0.005*
Triglycerides (mg/dL)	150 ± 25	180 ± 20	210 ± 35	240 ± 30	<0.001*
C-reactive Protein (mg/L)	1.5 ± 0.5	2.0 ± 0.6	3.0 ± 0.8	4.0 ± 0.9	<0.001*
Comorbidities (e.g., Hypertension, Obesity)	5 (20.8%)	10 (27.8%)	12 (35.3%)	15 (57.7%)	0.004*

## Discussion

The current study aimed to explore the relationship between HbA1c levels and the severity of CAD in patients presenting with ACS. Current study results indicate a strong association between higher HbA1c levels and the severity of coronary lesions, with a clear emphasis on increased prevalence of TVD and complex Type C lesions in patients with poorer glycemic control. Results had a good correlation with existing literature and pointed out more insights into the role of hyperglycemia, even at the non-diabetic range CAD progression. Similar to Girdhar et al. [13], the present study registered a higher incidence of multi-vessel disease among diabetic patients than their non-diabetic counterparts, which further supported the hypothesis that poor glycemic control worsens the course of CAD. The distribution of data indicated that higher values of HbA1c ≥10.5% are associated with a higher risk of severe coronary involvement, as shown by the 41.7% incidence of TVD. This corresponds well to the results of several earlier studies that have shown a strong relationship between HbA1c levels and the complexity of CAD. For instance, Ikeda et al. [14] observed that with rising HbA1c values, the severity of the coronary lesions was also enhanced in nondiabetic adults so that chronic hyperglycemia interacts with the cardiovascular system.

In addition, our study has shown that patients with HbA1c levels ranging from 6.5 to 8.5% also had significant coronary artery disease, and a significant number of them presented as DVD. This finding is in congruence with Vora et al. [15], who showed that more elevated HbA1c values in ACS patients are associated with more severe CAD severity and that there is an implication of some degree of glucose control to reduce cardiovascular risk. It is a testament to the strength of the association that the severity of coronary artery disease correlates so well with HbA1c, a level at which it can be added to the repertoire as a useful prognostic marker in patients with ACS, both with and without diabetes. Advanced glycation end-products (AGEs) are compounds formed when proteins or fats combine with sugars in the bloodstream, playing a significant role in the development of atherosclerosis through several mechanisms. AGEs promote oxidative stress and inflammation, leading to endothelial dysfunction, which is a critical precursor to CAD. Additionally, AGEs can enhance the stiffness of blood vessels and alter lipid metabolism, contributing to plaque formation and instability. Elevated levels of HbA1c signify chronic hyperglycemia, resulting in the accumulation of AGEs, which further promotes endothelial dysfunction, inflammation, and accelerated atherosclerosis.

A striking finding was that a large proportion of elevated HbA1c levels were associated with Type C lesions, the most complex and grave lesion type. This association aligns with another study by Won et al. [16], which demonstrated that greater serum AGE concentrations were predictive of CAD in diabetic subjects, independent of arterial stiffness. The development of AGEs under chronic hyperglycemic conditions is well recognized to enhance vascular stiffness and induce atherosclerosis, thereby contributing to the manifestations of more complex coronary lesions. Previous studies have shown that elevated levels of AGEs are associated with increased cardiovascular events, particularly in patients with diabetes, highlighting the need for careful glycemic control to minimize their formation and subsequent impact on vascular health. Moreover, the average duration of T2DM among the known diabetics in this study was 8.5 ± 3.2 years, and the average duration of CAD was 4.7 ± 2.1 years. Similar conclusions were drawn by Bhubaneshwar et al. [17], where a high statistically significant relationship was established between increased HbA1c levels and the severity of coronary lesions in diabetic patients with ACS, thereby supporting the results of the current study. This finding underscores the critical role of HbA1c not only as a marker of long-term glycemic control but also as a significant predictor of the severity of CAD in ACS patients.

The clinical implications are profound, as tight glycemic management and routine monitoring may slow the development of atherosclerosis and lower the likelihood of severe forms of CAD, such as Type C lesions and TVD. This is particularly true for individuals with diabetes; however, the findings also suggest that those without diabetes who exhibit elevated HbA1c levels may benefit from more aggressive management of cardiovascular risk factors. Additionally, the inclusion of HbA1c values in risk stratification could enhance prognosis prediction in patients with coronary artery disease, even though coronary angiography is considered the gold standard for diagnosing and assessing CAD severity. This can facilitate earlier interventions and improve patient management strategies. Such insights emphasize the necessity for individualized management approaches tailored to patients with ACS, particularly diabetic individuals who could significantly benefit from rigorous HbA1c monitoring alongside angiographic evaluation.

In addition to HbA1c levels, the current study evaluated several key biochemical parameters that were significantly associated with the severity of CAD. Total cholesterol, LDL, HDL, triglycerides, and C-reactive protein levels were assessed across different severities of CAD. Patients with TVD showed the highest total cholesterol (230 ± 35 mg/dL), LDL (150 ± 25 mg/dL), and triglycerides (240 ± 30 mg/dL), while those with SVD and DVD had progressively lower levels. In contrast, HDL levels were inversely related to CAD severity, with TVD patients displaying the lowest values (35 ± 5 mg/dL). CRP, a marker of systemic inflammation, also correlated significantly with CAD severity, peaking in TVD patients (4.0 ± 0.9 mg/L), suggesting an inflammatory component in more advanced coronary disease. Furthermore, the prevalence of comorbidities such as hypertension and obesity increased with the severity of CAD, with 57.7% of TVD patients affected. These findings highlight the role of dyslipidemia, inflammation, and comorbidities in CAD progression, emphasizing the need for comprehensive management strategies that target both metabolic and inflammatory pathways to reduce cardiovascular risk in patients with ACS.

Furthermore, as observed in studies by Dar et al. [18] and Pan et al. [19], the prognostic value of HbA1c extends beyond mere diabetes management, as it has been linked to outcomes in non-diabetic patients presenting with ACS. These studies advocate for integrating HbA1c measurements into routine clinical practice, as they can provide critical information regarding both short-term and long-term cardiovascular risk, facilitating timely interventions to improve patient outcomes. Enhanced awareness of the relationships between HbA1c, diabetes duration, and CAD severity can lead to better-targeted therapies and a more comprehensive approach to cardiovascular health management. This study's findings highlight the necessity of further investigation into the pathophysiological mechanisms that underpin the association between HbA1c levels and CAD severity. Understanding these mechanisms could lead to more effective interventions aimed at reducing cardiovascular morbidity and mortality in both diabetic and non-diabetic populations, reinforcing the importance of glycemic control as a key component in the management of coronary artery disease.

Limitations 

However, this study has several limitations. The sample size was limited to a single center, which may restrict the generalizability of the findings to broader populations. Additionally, the cross-sectional design limits the ability to establish causality between HbA1c levels and the progression of CAD. Confounding factors such as the duration of diabetes, medication adherence, and lifestyle choices were not explored in depth, which could influence the results. Lastly, reliance on self-reported medical history may introduce recall bias, potentially affecting data accuracy.

## Conclusions

This investigation identifies a robust correlation between elevated HbA1c levels and the severity of coronary artery disease in patients presenting with ACS. Specifically, higher HbA1c levels were significantly associated with more complex coronary lesions, including triple vessel disease and Type C lesions, suggesting that poor glycemic control accelerates atherosclerotic progression. These findings emphasize the importance of rigorous HbA1c monitoring and management in both diabetic and non-diabetic individuals at risk for coronary artery disease. Furthermore, incorporating HbA1c levels into clinical risk assessment algorithms may enhance the prediction of coronary artery disease severity and facilitate the tailoring of treatment strategies. The optimization of glycemic control, in conjunction with established therapeutic approaches, could be instrumental in mitigating the burden of coronary artery disease within high-risk populations.
